# Automatic Calibration of Odometry and Robot Extrinsic Parameters Using Multi-Composite-Targets for a Differential-Drive Robot with a Camera

**DOI:** 10.3390/s18093097

**Published:** 2018-09-14

**Authors:** Shusheng Bi, Dongsheng Yang, Yueri Cai

**Affiliations:** Robotics Institute, Beihang University, Beijing 100191, China; ssbi@buaa.edu.cn (S.B.); ydsf16@buaa.edu.cn (D.Y.)

**Keywords:** odometry calibration, extrinsic calibration, differential-drive robot, monocular camera

## Abstract

This paper simultaneously calibrates odometry parameters and the relative pose between a monocular camera and a robot automatically. Most camera pose estimation methods use natural features or artificial landmark tools. However, there are mismatches and scale ambiguity for natural features; the large-scale precision landmark tool is also challenging to make. To solve these problems, we propose an automatic process to combine multiple composite targets, select keyframes, and estimate keyframe poses. The composite target consists of an aruco marker and a checkerboard pattern. First, an analytical method is applied to obtain initial values of all calibration parameters; prior knowledge of the calibration parameters is not required. Then, two optimization steps are used to refine the calibration parameters. Planar motion constraints of the camera are introduced in these optimizations. The proposed solution is automatic; manual selection of keyframes, initial values, and robot construction within a specific trajectory are not required. The competing accuracy and stability of the proposed method under different target placements and robot paths are tested experimentally. Positive effects on calibration accuracy and stability are obtained when (1) composite targets are adopted; (2) two optimization steps are used; (3) plane motion constraints are introduced; and (4) target numbers are increased.

## 1. Introduction

Odometry and monocular camera have been widely used in indoor mobile robots owing to low cost and rich information. Fusing these two sensors for robot navigation is a popular research topic [1,2,3]. The operation of the fusion system requires odometry parameters, robot extrinsic parameters, and camera intrinsic parameters as prior knowledge. First, the odometry parameters usually refer to the structural parameters of the robot, such as wheel spacing, wheel radius, encoder line number, reduction ratio, and so on. These parameters can be roughly obtained from the design drawings of the robot or by manual measurements. However, in practical applications, the actual parameters are different from the design parameters due to manufacturing errors, non-point contact between the tire and the ground, changes in tire pressure, and load changes. Therefore, odometry calibration is required before the robot system works. Second, the robot extrinsic parameters refer to the relative pose between the camera and the robot. They are difficult to determine through the design drawings or manual measurements because of that the optical centre of the camera is a virtual point or that there are limitations of physical measurement tools. Accordingly, a robot extrinsic parameters calibration method is necessary. Third, the problem of camera intrinsic calibration has been studied extensively. Thus, this paper mainly considers the first two problems, namely simultaneously calibrating odometry and robot extrinsic parameters automatically.

Odometry errors can be categorized into two types: systematic and non-systematic errors [4]. The systematic error is mainly caused by inaccurate kinematic parameters, such as imprecise wheel radius. The non-systematic error is caused by robot environment interactions, such as wheel-slippage and uneven floors. The origination of most odometry errors has been discussed in [5]. Systematic errors are the primary source of odometry errors on smooth indoor surfaces [6]. Therefore, the odometry calibration addressed in this paper focuses on estimating robot kinematic parameters. Several studies [6,7,8,9,10,11] have considered this issue and proposed solutions. Among these, the UMBmark method [6] is widely used. In the UMBmark method, a differential-drive robot was controlled to follow a square path, where the odometry was calibrated based on the error between the final pose and the predicted pose. A generalized method was proposed accordingly for an arbitrary trajectory in [7]. Another method is recursive filtering, wherein calibration parameters are added to the system state vector, and a Bayesian filter is used to estimate parameters [8,9]. This method uses an odometry model for state propagation while updating from an external pose sensor. The third approach is the nonlinear batch optimization, which minimizes errors originating from the odometry [10,11]. These methods all assume that at least one external sensor is used to measure the actual pose of the robot.

Determining the relative pose between the camera and mobile robot is referred to in this paper as robot extrinsic parameter calibration. The purpose is to identify the best pose transformation to connect the mobile robot trajectory and camera trajectory. Estimating the robot trajectory typically resorts to wheel odometry [12,13], where the odometry parameters must be known in advance. The camera trajectory is obtained using natural features, such as points and lines in the environment [12,13,14], or by using artificial landmark tools, such as aruco markers [15] and others [16]. The natural feature-based method requires sufficient reliable features in the environment, a condition that is difficult to guarantee in some indoor settings. Furthermore, scale information cannot be obtained from a monocular camera. It is also infeasible to create a large, accurate landmark tool to ensure calibration accuracy using the artificial landmark-based method.

In fact, if robot extrinsic parameters were known, then a camera could be used as an external sensor to measure the robot pose and facilitate odometry calibration. Similarly, if the odometry parameters were known, then the robot extrinsic parameters could be easily obtained. Odometry parameter calibration and robot extrinsic parameter calibration are chicken–egg problems, which some researchers have tried to solve simultaneously. The analytical method [17,18] is one type of solution. In [17], a 3D landmark tool was applied to calibrate the camera intrinsic parameters and estimate its trajectory. The odometry and robot extrinsic parameters were determined based on the single value decomposition (SVD) method. Another technique is the filter-based method, in which the odometry parameters, robot extrinsic parameters, and robot configurations combine to form a state vector [19,20]. The odometry model is commonly employed for state propagation while updates are derived from visual observations. Another frequently used method involves solving an optimization problem to jointly minimize errors arising from integrated odometry measurements and (reprojection) errors in visual terms [15,21]. Such optimization approach offers the advantage of repeated linearization of the inherently nonlinear cost terms, which limits linearization errors. However, the filter-based method and optimization-based method each require good initial values for calibration parameters. By contrast, the analytical approach can provide calibration results without initial assumptions, but the result possesses lower accuracy. Therefore, an automatic calibration method was developed by combining the analytical method with an optimization-based method [15].

In this paper, an automatic method is proposed to calibrate the odometry and robot extrinsic parameters simultaneously. This approach automatically selects keyframes, calculates initial parameter values, and has no constraints on the robot path. First, we use multiple specially designed composite targets. Then, a new approach is introduced to combine these composite targets, select keyframes, and estimate the keyframe poses. Next, we use an analytical method to obtain initial values of the calibration parameters. Finally, two optimization steps are applied to determine the refined parameters. In addition, planar motion constraints are introduced into the optimization functions.

The remainder of this paper is organized as follows. In Section 2, we specify the problem. In Section 3, the proposed method is described in detail. A series of experiments are presented in Section 4. Finally, we offer conclusions and discussions in Section 5.

## 2. Preliminaries

**Figure 1:**
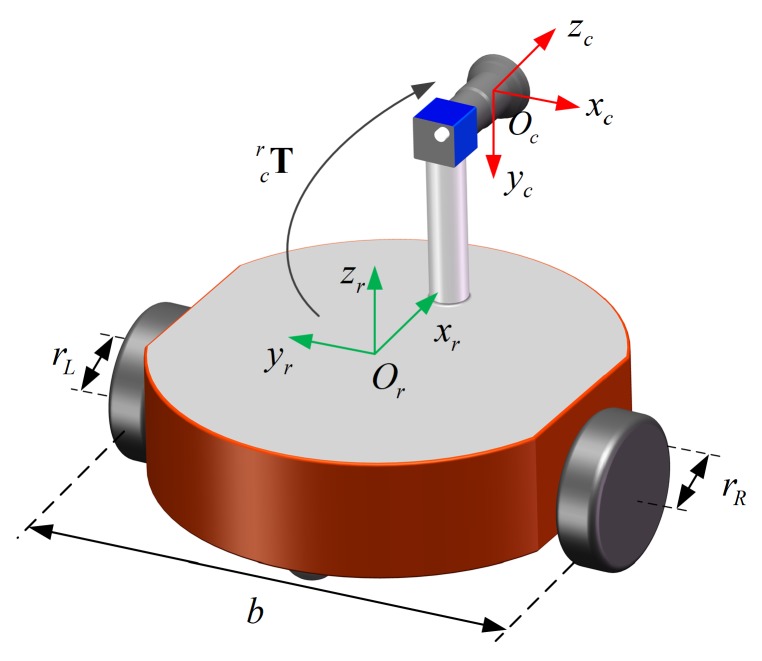
Schematic of a differential-drive mobile robot equipped with a monocular camera.

The system of a differential-drive mobile robot equipped with a monocular camera is shown in Figure 1. $r_{L}$ and $r_{R}$ are the left and right wheel radiuses. *b* is the wheel spacing. Two coordinate frames are established, namely, the robot coordinate frame {r} that is fixed to the robot, and the camera coordinate frame {c} that is fixed to the monocular camera. The robot is assumed to move on a two-dimensional (2D) plane. The $z_{r}$-axis of {r} is perpendicular to the plane. The $x_{r}$-axis of {r} is pointed to the front of the robot. The origin of {r} is set at the midpoint of the wheel axis.

### 2.1. Odometry Model

The pose of a mobile robot moving on a 2D plane can be expressed as ${\left[x,\;y,\;\theta \right]}^{T}$, where ${\left[x,\;y\right]}^{T}$ represents the translation movement, and θ represents the rotation angle. The robot pose at time point *k* is represented by $\left[x_{k},\;y_{k},\;\theta_{k}\right]$, then the robot pose at the next time point k+1 is given by the odometry model [22]:

(1) $$\left\{\begin{array}{c} x_{k+1}=x_{k}+\frac{k_{R}s_{R_{k}}+k_{L}s_{L_{k}}}{2}cos\left(\theta_{k}+\frac{k_{R}s_{R_{k}}-k_{L}s_{L_{k}}}{2b}\right),\\ y_{k+1}=y_{k}+\frac{k_{R}s_{R_{k}}+k_{L}s_{L_{k}}}{2}sin\left(\theta_{k}+\frac{k_{R}s_{R_{k}}-k_{L}s_{L_{k}}}{2b}\right),\\ \theta_{k+1}=\theta_{k}+\frac{k_{R}s_{R_{k}}-k_{L}s_{L_{k}}}{b}, \end{array}\right.$$

where $s_{L_{k}}$ and $s_{R_{k}}$ are encoder increments generated by the left encoder and the right encoder, respectively, between the time point *k* and k+1; and $k_{L}$ and $k_{R}$ are the left wheel factor and right wheel factor, respectively. The wheel factors transform encoder increments in the unit tick to wheel displacements in the unit m. The purpose of the odometry calibration is to obtain the precise wheel factors $k_{L}$ and $k_{R}$ as well as the precise wheel spacing *b*.

### 2.2. Camera Model

Many different camera models have been proposed in the literature [23,24]. A universal symbol π is used to represent the camera model that projects a three-dimensional (3D) point pc in the camera coordinate frame {c} to a 2D image coordinate $u={\left[u,\;v\right]}^{T}$:

(2) u=πpc.

In addition, the camera intrinsic parameters are assumed to be carefully pre-calibrated.

### 2.3. Robot Extrinsic Parameters

The robot extrinsic parameters to be calibrated represent the relative pose between the camera coordinate frame {c} and the robot coordinate frame {r}. They are expressed by a 4 × 4 homogeneous transformation matrix Tcr as shown Figure 1. To facilitate subsequent derivation, the ZYZ Euler angles $\alpha_{1}$, $\alpha_{2}$, and $\alpha_{3}$ are used to represent the rotation: Rcr=Rotz(α1)Roty(α2)Rotz(α3); and $t_{x}$, $t_{y}$, and $t_{z}$ are used to represent the translation: tcr=[tx,ty,tz]T. As the differential-drive robot moves on a 2D plane, there is no translation component in the $z_{r}$-axis direction; thus, $t_{z}$ is unobservable [13], which has been proved by [9]. This means that $t_{z}$ cannot be obtained by calibration. We set it to be 0. In summary, the robot extrinsic parameters to be estimated are $\alpha_{1}$, $\alpha_{2}$, $\alpha_{3}$, $t_{x}$ and $t_{y}$ in this paper.

In short, eight parameters must be calibrated in total: $k_{L}$, $k_{R}$, *b*, $\alpha_{1}$, $\alpha_{2}$, $\alpha_{3}$, $t_{x}$ and $t_{y}$.

## 3. Automatic Calibration Solution

The proposed automatic calibration method is discussed in this section. First, the main ideas and a calibration pipeline are introduced. Then, four key steps in the solution are explained.

### 3.1. System Overview

**Figure 2:**
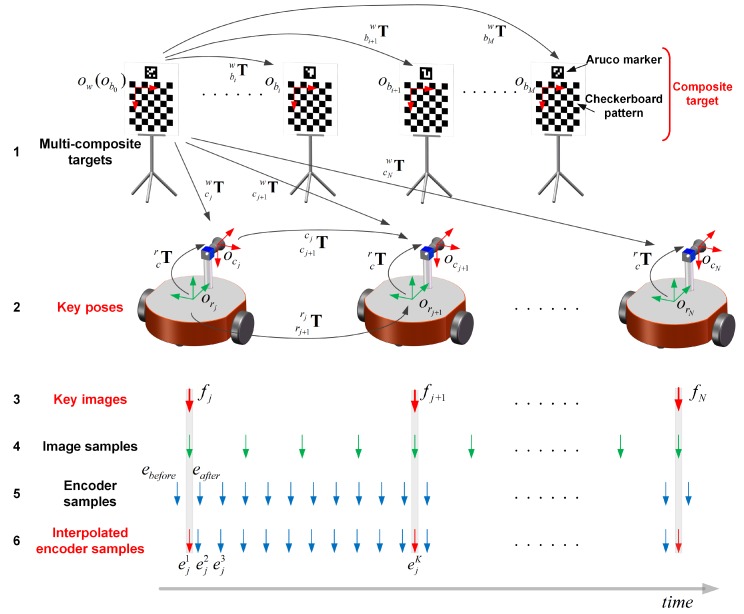
Schematic of automatic calibration method.

The proposed method is illustrated in Figure 2. Several specially designed composite targets are placed in the environment. The composite target consists of an aruco marker [25] and a checkerboard pattern. The checkerboard pattern provides a number of precise corners; these corners are used for camera poses and target poses estimation. The aruco marker can provide an independent ID, which is used to avoid mismatches. Commonly, multiple targets may be captured in one image. Checkerboard corners of each target should be extracted separately in the image. To address this problem, we have two conditions at hand. First, we assume that the target boundary positions relative to the aruco marker are pre-known, which can be obtained from the target design parameters. Second, the aruco marker can provide the relative pose between the camera and the aruco marker [25]. Then, the target boundaries can be projected to the image, and the image area of the target is obtained. Thus, the checkerboard corners of this target can be extracted from this image area.

Multiple composite targets are used to improve the accuracy of camera pose estimation, thereby enhancing calibration accuracy. It is difficult for natural feature-based methods to achieve high precision camera pose estimation due to mismatching, scale problems for the monocular camera, or a lack of features in the environment. Commonly used artificial landmarks provide few corner points and suffer from low precision problems, such as binary square fiducial markers. The composite target proposed in this paper can produce many accurate corner points via the checkerboard. Using an aruco ID also avoids mismatches. However, a small size target or landmark tool only covers part of the image, resulting in low camera pose estimation accuracy. Moreover, a high precision, large target or landmark tool is difficult to produce, has poor portability, and is costly. Given these drawbacks, multiple targets are employed here.

After the targets are laid out, the robot is controlled to move arbitrarily in front of them. The encoders and image data with timestamps are recorded. The layout of the targets and the robot trajectory are not limited, but for the precision considerations, the robot is recommended to move slowly to avoid wheel slippage. Additionally, as many targets as possible should be within the field of the camera.

Some key images (the third row of Figure 2) are selected carefully, rather than using all the images. The key images are also called keyframes. The camera pose corresponding to the keyframe is called the key camera pose or keyframe pose. The keyframe is used for two reasons: first, the difference between two adjacent image samples is too small and will cause a pathological state in the analytical solution as described in Section 3.3; second, using all images will result in a sharp increase in the amount of computation, which renders a solution challenging.

i∈[1,M] indicates the number of the target, j∈[1,N] indicates the number of the keyframe, and k∈[1,K] indicates the number of the encoder sample between two adjacent keyframes. A target coordinate $\left\{b_{i}\right\}$ is established for every target. The world coordinate frame {w} coincides with the target coordinate frame of the first observed target. The camera coordinate frame $\left\{c_{j}\right\}$ and the robot coordinate frame $\left\{r_{j}\right\}$ are established corresponding to the *j*th keyframe. The relative pose of the robot between two adjacent keyframes is expressed by a 4 × 4 homogeneous transformation matrix Trj+1rj, and the relative pose of the camera is expressed by Tcj+1cj correspondingly. The camera pose in the world coordinate frame is expressed by Tcjw. The target pose in the world coordinate frame is expressed by Tbiw.

Generally, the encoders and the camera form an asynchronous acquisition system. Typically, no encoder sampling occurs at the time of the keyframe (compare the third and the fifth rows in Figure 2) because the sampling frequency of the encoder is higher than that of the camera. Therefore, the linear interpolation method is used to produce an encoder sample corresponding to the *j*th keyframe with the previous encoder sample $e_{before}$ before the *j*th keyframe and the next one $e_{after}$ after the *j*th keyframe (see the fifth and the sixth rows in Figure 2), such as $e_{j}^{1}$ in Figure 2:

(3) $$e_{j}^{1}=\frac{t_{j}-t_{e_{before}}}{t_{e_{after}}-t_{e_{before}}}\left(e_{after}-e_{before}\right)+e_{before},$$

where *t* with a right subscript represents the time corresponding to the *j*th keyframe or encoder samplings $e_{before}$ and $e_{after}$.

The calibration process can be divided into the following four steps:

- Step 1: an automatic pipeline is designed to combine the composite targets, select the keyframes and estimate the keyframe poses.
- Step 2: an analytical method is used to solve the initial values of the calibration parameters.
- Step 3: an optimization problem is built by minimizing odometry error terms to refine the calibration parameters.
- Step 4: a total optimization containing all error terms is constructed to obtain the final optimized calibration parameters.

These four steps are discussed in detail in subsequent sections.

### 3.2. Estimation of Keyframe Poses

**Figure 3:**
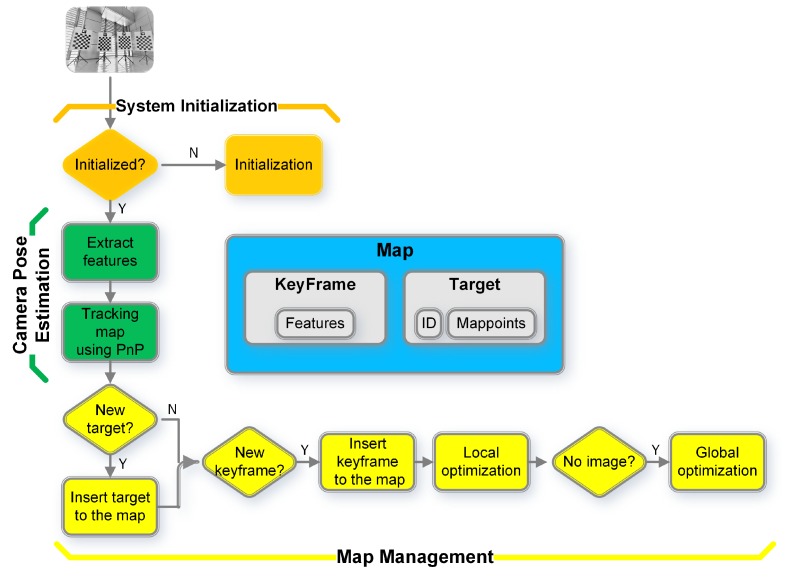
Process of keyframe poses estimation.

This step aims to select keyframes and to estimate each keyframe pose $wc_{j}T$ by integrating multiple targets. The recorded images are extracted and processed according to the processing flow shown in Figure 3. The flow is divided into four parts: map, system initialization, camera pose estimation, and map management.

#### 3.2.1. Map

The map is used to maintain a series of keyframes and targets. Checkerboard corner points on each target build a cluster of 3D landmark points, referred to as map points. The positions of the map points relative to the target coordinate frames can be known in advance according to the structure of the targets. Each target has an aruco marker ID to avoid false matches. The 2D corners of one target in the image are called features. The collocation of a key image (see the third row of Figure 2) and extracted 2D features form a keyframe.

#### 3.2.2. System Initialization

When the first image arrives, the first target observed by the image will be added to the map. The world coordinate frame {w} coincides with the coordinate frame $\left\{b_{0}\right\}$ of the first target. Correspondingly, the first image is constructed as a keyframe: the feature points are extracted, and the coordinate frame relationship Tc0w between the world coordinate fame {w} and the camera coordinate frame $\left\{c_{0}\right\}$ is calculated using the perspective-n-point (PnP) method [26].

#### 3.2.3. Camera Pose Estimation

After initialization, the keyframe and the target exist in the map. The system then enters a normal process. When the *l*th image comes in, its pose will be estimated. Multiple targets may be observed in the image. The features of each observed target from the image will be extracted firstly. The corresponding targets are searched in the map using aruco IDs. After this, matches of 3D map points to 2D features are obtained. Then, the PnP method is used to get the camera pose Tclw. It needs to point out that there are multiple targets in the map in the normal process, and the coordinates of the map points of each target are unified to the world coordinate frame {w}.

#### 3.2.4. Map Management

Next, the *l*th image is checked to determine whether new targets exist, if so, they will be added to the map. First, the PnP method is used to calculate the homogeneous transformation Tbicl between the camera coordinate frame $\left\{c_{l}\right\}$ and the new target coordinate frame $\left\{b_{i}\right\}$. Then, the homogeneous transformation from the new target coordinate frame $\left\{b_{i}\right\}$ to the world coordinate frame {w} is achieved:

(4) Tbiw=TclwTbicl.

The map points on the new targets can be unified to the world coordinate frame.

Then, the selection of keyframes is introduced under two conditions. First, at least two targets must be observed by the image to ensure sufficient constraints between the keyframes and the targets to achieve higher accuracy in subsequent optimization steps. Second, the angle change and distance between the current image and the last keyframe should be greater than thresholds $\theta_{th}$ and $d_{th}$, where:

(5) $$\left\{\begin{array}{c} \theta_{th}=rand\left[\theta_{min},\;\theta_{max}\right],\\ d_{th}=rand\left[d_{min},\;d_{max}\right]. \end{array}\right.$$

The rand[] refers to the uniform sampling. The purpose of using random thresholds is to ensure that the angles and the displacements between keyframes are different to guarantee the correct solution for the analytical solve step (Section 3.3). When both above conditions are satisfied, the *l*th image is constructed as the *j*th keyframe and added to the map.

**Figure 4:**
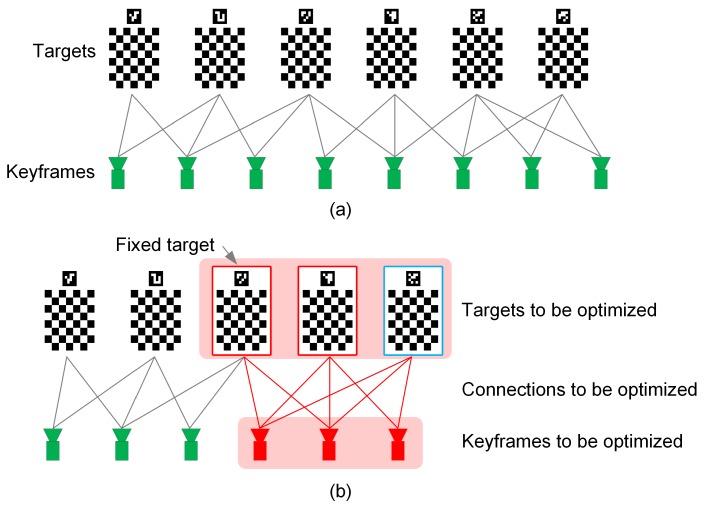
Networks between targets and keyframes. (**a**) global network; (**b**) local network.

When a target is observed in a keyframe, a line in Figure 4a is drawn to connect the keyframe with the target. More connections create a more stable network with more accurately estimated keyframe poses. Once all images in the recorded data have been processed, a global optimization is carried out:

(6) λ={Tb2w,Tb3w,⋯,TbMw;Tc1w,Tc2w,⋯,TcNw;A,B,C,D},λ*=argminλ∑i∈κ∑j∈ΘEproj(i,j)+∑j=1NEplanar(j)+∑j=1N−1Erot(j,j+1),

where κ denotes the set of all targets, Θ denotes the set of all keyframes. The optimization equation (Equation (6)) is composed of three parts. The first is the projection error from the map points to the features. For one connection between the *i*th target and the *j*th keyframe, the error term is defined as:

(7) Eproj(i,j)=∑q=1QeqTΣq−1eq,eq=uqj−πT−1cjwTbiwpqbi,

where *Q* is the number of corners in the target, and uqj is the *q*th 2D feature in the target extracted from the *j*th keyframe, pqbi is the *q*th 3D map point in the target expressed in the target coordinate frame $\left\{b_{i}\right\}$, and $\Sigma_{q}$ is the covariance matrix.

The second and the third parts are constraints generated by the planar motion of the camera. The second part considers the keyframe positions. The error term is defined as the square of the distance from the keyframe position tcjw:[txcjw,tycjw,tzcjw] to the motion plane Ax+By+Cz+D=0. For the *j*th keyframe:

(8) Eplanar(j)=∥Atxcjw+Btycjw+Ctzcjw+D∥2A2+B2+C2.

The third part considers the keyframe rotations. For a planar motion camera, the axes of rotations Rcj+1cj between adjacent keyframes are in the same direction. This direction is the normal vector of the motion plane $n:\;{\left[A,\;B,\;C\right]}^{T}$. Rcj+1cj is transformed to axis–angle representation rcj+1cj. Then, the rotation error is defined by the cross of n and rcj+1cj:

(9) Erot(j,j+1)=∥n×rcj+1cj∥2.

The initial values of *A*, *B*, *C*, and *D* are obtained via data fitting using all keyframe positions. Tb0w is not optimized because the coordinate frame $\left\{b_{0}\right\}$ of the first target coincides the world coordinate frame {w}.

Good initial values are necessary for global optimization. However, the keyframe and the target insertion process can generate cumulative errors, resulting in low precision of initial poses of keyframes and targets. To address this problem, a local optimization process is introduced to reduce error accumulation. The local optimization is performed when a newly inserted target (the target with the blue edge in Figure 4b) is observed by more than $N_{th}$ keyframes. The local optimization involves a set {s} of keyframes that connected directly to the target (those red ones in Figure 4b), and targets that directly connected to the keyframe set {s} (those with the red board in Figure 4b), and the connections between them. Different from the global optimization, only the projection errors are minimized in the local optimization, and the oldest target is fixed.

In this step, the precise poses of *M* targets, the poses of *N* keyframes, and the connection relationships between them are obtained. Next, an analytical solution will be used to obtain the initial values of the calibration parameters.

### 3.3. Estimating Initial Values of Calibration Parameters

In this step, a modified analytical method [17] is used to estimate the initial values of the calibration parameters. The difference is that the coordinate frames vary slightly, which results in variations in the derivation. Considering the relative pose changes between the *j*th keyframe and the (j+1)th keyframe in Figure 2, we obtain the following:

(10) TcrTcj+1cj=Trj+1rjTcr.

Decomposing Equation (10) into rotation and translation forms:

(11) RcrRcj+1cj=Rrj+1rjRcr,Rcrtcj+1cj+tcr=Rrj+1rjtcr+trj+1rj.

The rotation matrix Rcj+1cj is converted into the axis–angle representation $r=\hat{r}\cdot \Delta \theta_{j+1}^{j}$. $\hat{r}=\left[r_{x},\;r_{y},\;r_{z}\right]$ is the normalized axis, and $\Delta \theta_{j+1}^{j}$ is the rotation angle. The rotation of the rigid body keeps uniform throughout, thus, $\hat{r}$ should be in the same direction as the $z_{r}$-axis of the robot coordinate frame $\left\{r_{j}\right\}$. Then, $\alpha_{2}$ and $\alpha_{3}$ are given [27] by:

(12) $$\left\{\begin{array}{c} \alpha_{2}=atan2\left(\sqrt{r_{x}^{2}+r_{y}^{2}},\;r_{z}\right),\\ \alpha_{3}=atan2\left(r_{y},\;-r_{x}\right). \end{array}\right.$$

Because the robot moves on a 2D plane and the $z_{r}$-axis is perpendicular to this plane, the robot rotation change Rrj+1rj between the *j*th keyframe and the (j+1)th keyframe is only a rotation around the $z_{r}$-axis: Rrj+1rj=Rotz(Δθj+1j). The rotation angle $\Delta \theta_{j+1}^{j}$ can also be calculated by the odometry model (Equation (1)) with an initial value of 0:

(13) $$\beta_{R}\sum_{k=1}^{K-1}s_{R_{k}}-\beta_{L}\sum_{k=1}^{K-1}s_{L_{k}}=\Delta \theta_{j+1}^{j},$$

(14) $$\left\{\begin{array}{c} \beta_{L}=\frac{k_{L}}{b},\\ \beta_{R}=\frac{k_{R}}{b}, \end{array}\right.$$

where *K* is the number of encoder samples between two adjacent keyframes; and $\beta_{L}$ and $\beta_{R}$ are intermediate variables. Thus, N−1 Equation (13) can be derived using *N* keyframes. If N>3, an overdetermined equation is formed:

(15) $$A{\left[\beta_{L}\;\beta_{R}\right]}^{T}=b,$$

which can be solved by the least square method:

(16) $${\left[\beta_{L}\;\beta_{R}\right]}^{T}={\left(A^{T}A\right)}^{-1}A^{T}b.$$

If all $\Delta \theta_{j+1}^{j}$ are equal, then the function is numerically poor. Therefore, the random threshold of the angle is used in Section 3.2.4 to avoid this problem.

Consider the second line of Equation (11). As $sin(\alpha_{1})$ and $cos(\alpha_{1})$ introduce nonlinear terms in Rcr, two intermediate variables: $s1=sin(\alpha_{1})$, $c1=cos(\alpha_{1})$ that are constrained by $s1^{2}+c1^{2}=1$ are introduced. Because tcr only has two degrees of freedom, only two functions are provided by the second row of Equation (11):

(17) $$\left\{\begin{array}{c} a_{1}s1+a_{2}c1+a_{3}k_{L}+a_{4}t_{x}+a_{5}t_{y}=0,\\ b_{1}s1+b_{2}c1+b_{3}k_{L}+b_{4}t_{x}+b_{5}t_{y}=0, \end{array}\right.$$

where a1−a5 and b1−b5 are known coefficients that derived from the second line of Equation (11). Then, N−1 Equation (17) are obtained by *N* keyframes , forming a overdetermined equation when N>4:

(18) $$\Omega \;{\left[s1\;c1\;k_{L}\;t_{x}\;t_{y}\right]}^{T}=0.$$

With the proper choice of keyframes (as in Section 3.2.4), Rank(Ω)=4 [17]. Thus, Ω has a one-dimensional null space. To solve Equation (18), we decompose Ω by the SVD method:

(19) $$\Omega =U\Sigma V^{T},$$

where U is a 2(N−1)×2(N−1) unitary matrix; Σ is a 2(N−1)×5 rectangular diagonal matrix with five non-negative singular values listed in decreasing order along the main diagonal. V is a 5×5 unitary matrix whose columns are right-singular vectors. Thus, the fifth column of V, $v_{5}$, spans the null space of Ω. Accordingly, the general solution of Equation (18) is given by:

(20) $$\varsigma^{*}=\eta v_{5},$$

where η is a constant factor. Under the constraint of $s1^{2}+s2^{2}=1$, η can be determined by

(21) $$\eta =\frac{1}{\sqrt{v_{5,1}^{2}+v_{5,2}^{2}}},$$

where $v_{5,1}$ and $v_{5,2}$ are the first and the second elements of $v_{5}$. Then, s1, c1, $k_{L}$, $t_{x}$, and $t_{y}$ are solved. Thus,

(22) $$\alpha_{1}=atan2\left(s1,c1\right),\;\;k_{R}=\frac{\beta_{R}}{\beta_{L}}k_{L},\;\;b=\frac{k_{L}}{\beta_{L}}.$$

Clearly, $\alpha_{2}$ and $\alpha_{3}$ calculated using the analytical method only requires the information of two keyframes and can thus easily experience noise interference. The condition of Rank(Ω)=4 is also easily affected by noises and does not hold. The least squares and SVD methods do not consider the difference in observation noises. The above problems reduce the accuracy of the analytical method. Tacking the analytical solutions as the initial values, two optimization steps are designed to obtain more accurate calibration parameters as follows.

### 3.4. Optimization of Calibration Parameters

In this step, only the calibration parameters are optimized by minimizing the odometry observation errors:

(23) $$\begin{array}{c} \lambda =\{k_{L},\;k_{R},\;b,\;\alpha_{1},\;\alpha_{2},\;\alpha_{3},\;t_{x},\;t_{y}\},\\ \lambda^{*}=\underset{\lambda }{\arg\min}\sum_{j=1}^{N-1}E_{odom}\left(j,\;j+1\right). \end{array}$$

The error term is calculated between two adjacent keyframes: the *j*th and (j+1)th keyframe. It is designed as the difference between the robot pose change calculated by the odometry and the robot pose change derived from the keyframe poses:

(24) Eodom(j,j+1)=eodom(j,j+1)TΣodom(j,j+1)−1eodom(j,j+1),eodom(j,j+1)=logTrj+1rj−logTcr(Tcjw)−1Tcj+1w(Tcr)−1,

where the log() operator transforms a 4 × 4 homogeneous transformation matrix into a six-dimensional column vector that containing three ZYZ Euler angles and three translation elements. $\Sigma_{odom(j,\;j+1)}$ is the covariance matrix with the odometry observation. Next, we demonstrate how to obtain the covariance matrix. The left/right real encoder increments between two encoder samples is assumed to obey a Gaussian distribution [22]:

(25) $$\hat{s_{L/R}}∼N\left(s_{L/R},\;K_{L/R}\left|s_{L/R}\right|\right).$$

The mean is calculated by the two encoder readings, and the left/right variance is proportional to the absolute value of the increment. Assuming that the 3 × 3 covariance of the robot pose at the time point of the *k*th encoder is $\Sigma_{o,k}$, then the robot pose covariance of the next moment k+1 is given by the linear error deduction method [22]:

(26) $$\Sigma_{o,k+1}=G_{o}\Sigma_{o,k}G_{o}^{T}+G_{e}\Sigma_{e,k}G_{e}^{T},$$

where $G_{o}$ is the Jacobian matrix of Equation (1) vs. the robot pose ${\left[x_{k},\;y_{k},\;\theta_{k}\right]}^{T}$; $G_{e}$ is the Jacobian matrix of Equation (1) vs. the encoder displacement ${\left[s_{L_{k}},\;s_{R_{k}}\right]}^{T}$; and $\Sigma_{e,k}$ is the covariance of the left and the right encoder displacements, according to Equation (25):

(27) $$\Sigma_{e,k}=\left[\begin{array}{cc} K_{L}\left|s_{L_{k}}\right| & 0\\ 0 & K_{R}\left|s_{R_{k}}\right| \end{array}\right].$$

If there are *K* encoder samples between the *j*th keyframe and the (j+1)th keyframe, the robot pose covariance at time point of the (j+1)th keyframe is obtained by iteratively using the Equation (26) with initial robot pose covariance $\Sigma_{o,1}=0$:

(28) $$\Sigma_{o,K}=\left[\begin{array}{ccc} \sigma_{x,x} & \sigma_{x,y} & \sigma_{x,\theta }\\ \sigma_{y,x} & \sigma_{y,y} & \sigma_{y,\theta }\\ \sigma_{\theta ,x} & \sigma_{\theta ,y} & \sigma_{\theta ,\theta } \end{array}\right].$$

It is the odometry observation covariance between the two adjacent keyframes. It should be noted that the odometry observation covariance used in Equation (24) is a 6 × 6 matrix. To ensure the establishment of Equation (24), the variances of the other two angles are assumed to be the same as θ, and the translational variance of *z* is assumed to equal to the minimum of variances of *x* and *y*:

(29) $$\Sigma_{odom(j,\;j+1)}=\left[\begin{array}{cccccc} \sigma_{\theta ,\theta } & 0 & 0 & 0 & 0 & 0\\ 0 & \sigma_{\theta ,\theta } & 0 & 0 & 0 & 0\\ 0 & 0 & \sigma_{\theta ,\theta } & \sigma_{\theta ,x} & \sigma_{\theta ,y} & 0\\ 0 & 0 & \sigma_{x,\theta } & \sigma_{x,x} & \sigma_{x,y} & 0\\ 0 & 0 & \sigma_{y,\theta } & \sigma_{y,x} & \sigma_{y,y} & 0\\ 0 & 0 & 0 & 0 & 0 & min\{\sigma_{x,x},\;\sigma_{y,y}\} \end{array}\right].$$

### 3.5. Final Optimization

Lastly, a total optimization is performed to minimize all errors. Ceres Solver [28] is used to solve all optimization problems in this paper:

(30) λ={kL,kR,b,α1,α2,α3,tx,ty;Tb2w,Tb3w,⋯,TbMw;Twc1,Twc2,⋯,TwcN;A,B,C,D},λ*=argminλ∑j=1N−1Eodom(j,j+1)+∑i∈κ∑j∈ΘEproj(i,j)+∑j=1NEplanar(j)+∑j=1N−1Erot(j,j+1).

## 4. Experiments

Several experiments were performed to verify the accuracy and the stability of the proposed method and to test the effect of four strategies: the adoption of multiple composite targets, the use of two optimization steps, the introduction of planar constraints, and the increases of the number of targets.

### 4.1. Experimental Setup

**Figure 5:**
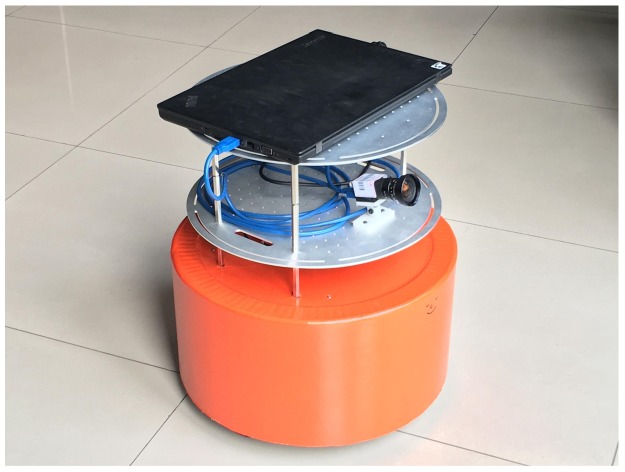
Redbot with a daheng monocular camera.

Figure 5 displays the Redbot used in our experiments. The Redbot is a small differential-drive mobile robot. The nominal left/right wheel radius $r_{L/R}$ is 0.05 m, the wheel spacing *b* is 0.32 m, the reduction ratio of the left/right motor is 7.2:1, and the encoder produces 1024 ticks per motor round with the frequency of 100 Hz. A daheng MER-302-56U3M/C camera with a Kawa LM3NCM wide-angle lens (Torrance, CV, USA) was installed. The camera resolution was set to 2048 × 1536 pixels, and the frame rate was set to 10 Hz. A Thinkpad laptop collected the encoder readings and images with a rosbag tool [29] provided in the Robot Operating System (ROS) [30]. The rosbag tool can record multiple types of data with timestamps. The checkboard pattern size is 6 × 7, and its grid size is 0.07 m. The side length of the aruco marker is 0.14 m. Composite targets were printed on A1-sized papers and attached to 15 mm thick Polyvinyl chloride (PVC) boards.

### 4.2. Performance Test

**Figure 6:**
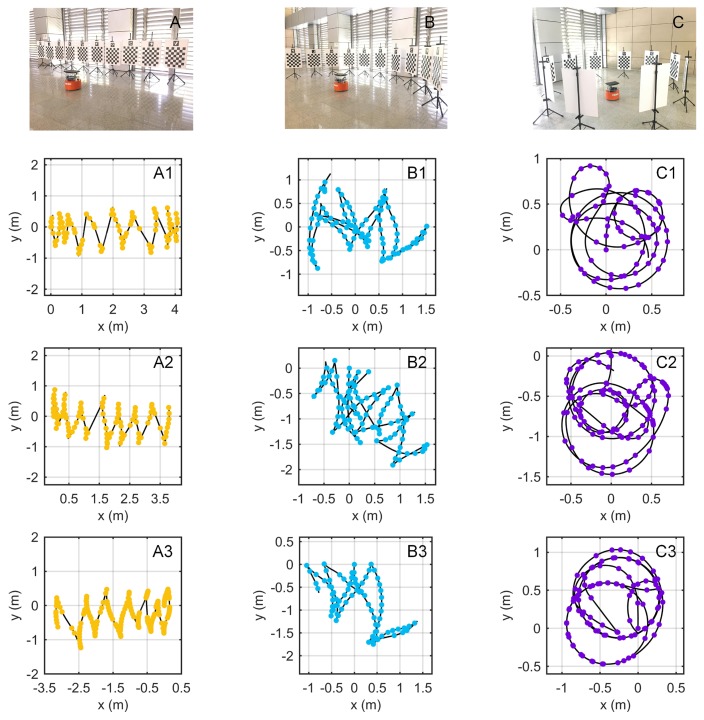
Experimental setup for the performance test. Ten targets were arranged in three forms: A, B, and C. Three data were recorded for each placement: data A1, A2, and A3 for form A, data B1, B2, and B3 for form B, and data C1, C2, and C3 for form C. Dots indicate selected keyframes, and lines indicate robot trajectories.

The purpose of this experiment was to test the stability of our method on different data. To verify the impact of the target placement form, as shown in Figure 6, ten targets were placed in three forms: A, B, and C. Then, the robot was controlled to move arbitrarily in front of the targets. For each placement form, three different data were recorded (e.g., A1, A2, and A3 for the placement form A).

To verify the method, three other variants were compared as follows. In the NoPlanar method, the planar motion error terms in Equations (6) and (30) were deleted to verify the role of the planar constraints. In the Analytical method, the last two optimization steps (Section 3.4 and Section 3.5) were removed to test the function of the last two optimization steps. In the Aruco method, the four corners of each aruco marker were used as map points instead of the corners of the checkerboard pattern, intended to prove the advantages of using composite targets.

**Figure 7:**
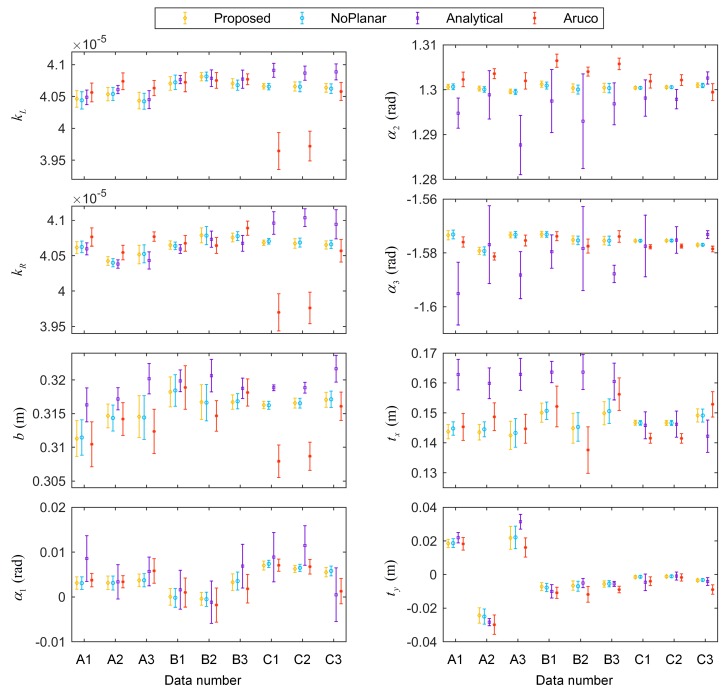
Results of calibration parameters with standard deviations.

Because the thresholds were random when selecting the keyframes, the result of each run of the algorithm could differ. For each data in Figure 6, each method was run ten times. The results of eight calibration parameters are shown in Figure 7, with the standard deviations of the ten runs.

The calibration results of different methods varied. In terms of the mean values of the results, relatively small fluctuations across different data appeared in the proposed method and the NoPlanar method because the two optimization steps improved the stability. Additionally, these two methods used the composite targets compared with the Aruco method. The composite targets contain many more precision map points; hence, these two methods achieved higher stability. The standard deviations represent the effect of keyframe selection. The standard deviations of the Analytical method were the largest given the absence of the two optimization steps. By contrast, the standard deviations of the proposed method and the NoPlanar method were relatively small compared with the Analytical method and the Aruco method, further confirming the positive effects of the two optimization steps and the composite targets. Moreover, the means and standard deviations of the proposed method and the NoPlanar method were similar, indicating that the introduction of planar constraints exerted little influence on the means and standard deviations of the calibration results; thus, they had little effect on calibration stability.

Some differences also emerged between different target placement forms in the proposed method. Figure 7 presents that the target placement form C achieved the highest stability, followed by B with A the worst. Moreover, the calibration results of placement forms B and C were similar. Figure 6 shows that the three trajectories of placement form A assumed open chain shapes; therefore, the networks built by the keyframes and targets were also open chains, resulting in unstable calibration results. On the contrary, the trajectories of placement form C were circles, and the networks assumed a closed form. Consequently, the calibration results were more stable than the other two target placement forms. The networks of placement form B were somewhere between A and C. Therefore, the targets should be arranged in a circular shape to obtain more stable calibration results.

Although we could obtain the mean and variance of the calibration parameters, we could not test the accuracy of the calibration results directly due to the absence of real calibration parameter values. To address this problem, two indirect experiments were designed to verify the calibration accuracy of the odometry and the external parameters of the robot.

### 4.3. Odometry Calibration Accuracy Test

**Figure 8:**
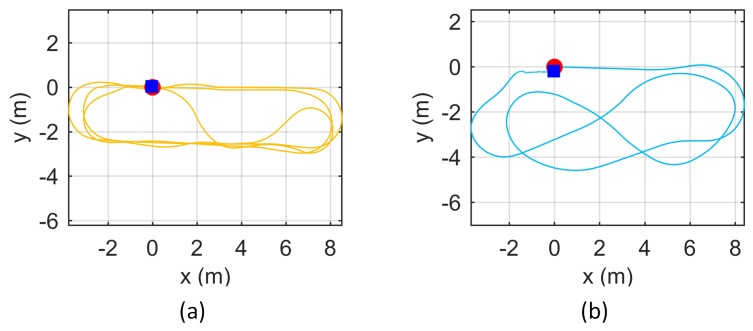
Robot trajectories of data (**a**,**b**). Red circles are starting points, and blue squares are the path ends; ideally, they should be coincident.

**Figure 9:**
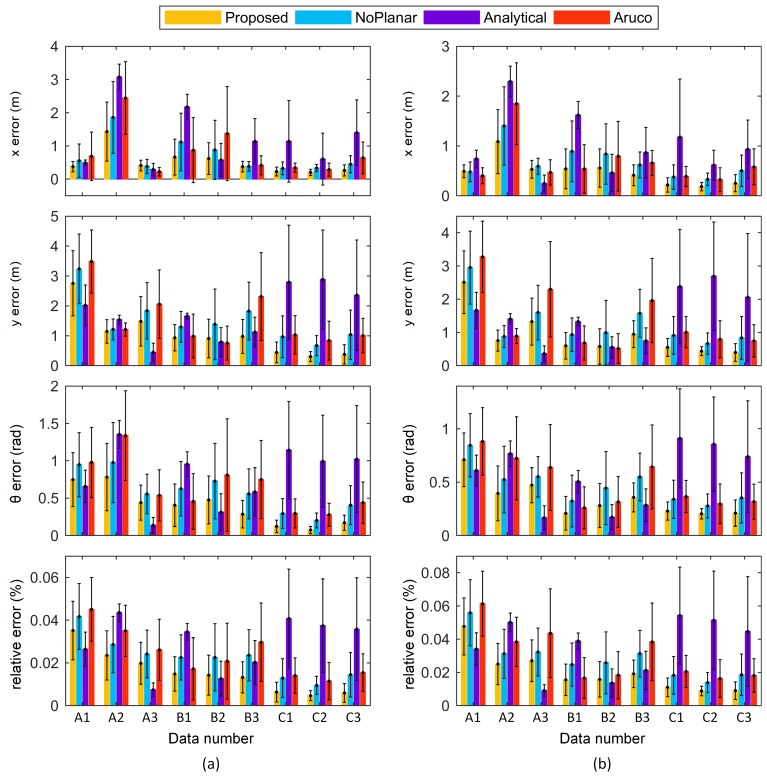
Odometry errors of different methods with different data using trajectories (**a**,**b**) from Figure 8.

If the odometry parameters are calibrated accurately, then the pose estimated by odometry will be accurate. An experiment was designed based on this assumption. The robot was controlled to run for a long distance and controlled back to the initial pose. Ideally, the end pose should be the same as the initial pose with a value of 0. However, due to errors in the odometry calibration, the two poses may be different. We used the endpoint error to indicate odometry calibration accuracy. Data were recorded in this experiment as shown in Figure 8. The lengths of these two trajectories were 79.6 m and 54.5 m. All calibration results in Section 4.2 were used to calculate the endpoint errors illustrated in Figure 9.

**Figure 10:**
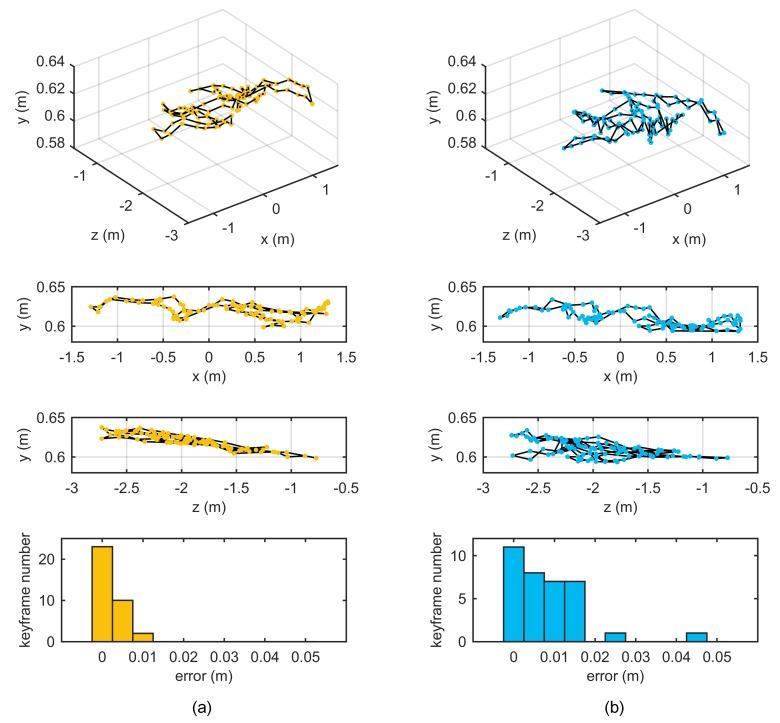
Comparison of keyframe positions between proposed method and NoPlanar method. (Axes scales are different): (**a**) proposed method; (**b**) NoPlanar method. The first row contains 3D keyframe positions. The second and third rows are side views of the first row. The last row is the distance histogram between the keyframe position and estimated move plane.

In most cases, the proposed method obtained the highest odometry accuracy due to using multiple composite targets, introducing planar constraints, and adopting two optimization steps. The odometry errors with calibration results from target placement form C were smaller than those from A and B, suggesting that the circular target placement form achieved higher odometry calibration accuracy. Although the means and standard deviations of the calibration results of the proposed and NoPlanar methods were similar, the proposed method achieved higher odometry calibration accuracy, potentially because the introduction of plane constraints improved the estimation accuracy of keyframe poses. Figure 10 shows keyframe position differences between the proposed method and the NoPlaner method with one data (B1). The robot moved on a plane, and the estimated keyframe positions should also be distributed on the same plane; however, keyframe positions estimated by the NoPlanar method were not on the same plane and demonstrated larger errors (see Figure 10b). Conversely, the proposed method introducing planar constraints obtained relative better keyframe position estimations (see Figure 10a).

### 4.4. Robot Extrinsic Calibration Accuracy Test

**Figure 11:**
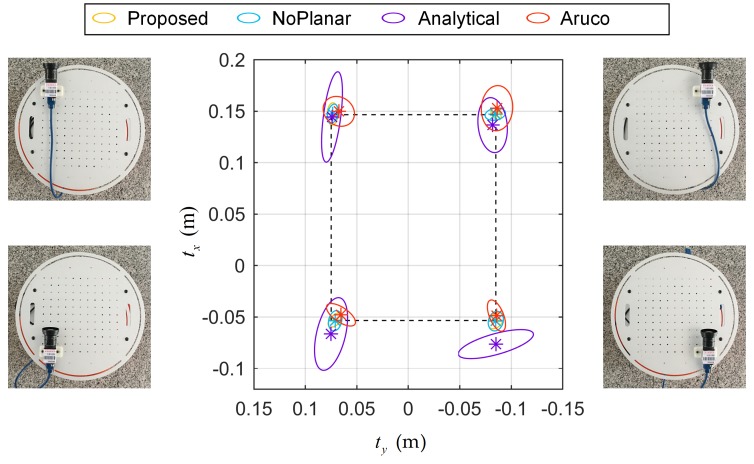
Robot extrinsic calibration precision test setup and results. ∗ denotes the mean of ${\left[t_{x},\;t_{y}\right]}^{T}$, the ellipse indicates covariance at 95% confidence, and the dotted rectangle is the nominal rectangle.

To test the accuracy of the robot extrinsic calibration, as shown in Figure 11, the camera was placed at four positions on the robot for calibration, and ten targets were arranged in a circle. These four positions formed a rectangle. The structure of the robot could determine the length (0.2 m) and width (0.16 m) of the rectangle. One data was collected per position. As in Section 4.2, each method was run ten times with the data. Ideally, greater similarity between the nominal rectangle and the rectangle formed by calibration results will lead to more accurate calibration results. As shown in Figure 11, the covariance ellipses with 95% confidence represent the distributions of ${\left[t_{x},\;t_{y}\right]}^{T}$ and the symbols ∗ indicate the means of ${\left[t_{x},\;t_{y}\right]}^{T}$. The nominal rectangle is shown in Figure 11 by dotted lines. Considering the mean values, the four corner points obtained by the proposed method and NoPlanar method were similar to the corner points of the nominal rectangle. However, the Analytical method and Aruco method demonstrated large gaps. For the covariance ellipse, the ellipse size of the proposed method was smaller than others, indicating that the composite target and the optimization steps used in this paper can improve the accuracy and stability of extrinsic parameter calibration. It was not possible to test absolute accuracy, but the relative accuracy was evaluated. Mean values were used to calculate the average length and width of the rectangle along with the average angle of the four angles. Then, they were compared with the nominal length, width, and angle to compute the relative errors. Results are listed in Table 1. Although only the angular error of the proposed method was smallest compared to other methods, the width and length errors of the proposed method were highly similar to the minimum errors. Unfortunately, the accuracy of $\alpha_{1}$, $\alpha_{2}$, and $\alpha_{3}$ could not be assessed effectively. Figure 7 reveals that the results of the proposed method with a target placement of C demonstrated small angle fluctuations within a range of less than 0.01 rad. Such stability can meet the needs of conventional applications. Overall, the robot extrinsic calibration accuracy and stability of the proposed method was relatively higher than others.

### 4.5. The Impact of the Number of Targets

**Figure 12:**
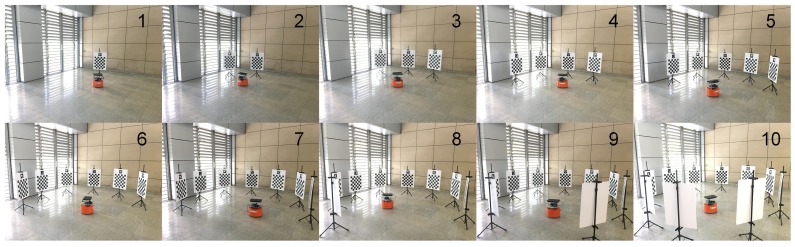
Experimental setup to test the effect of the number of the targets on calibration results.

**Figure 13:**
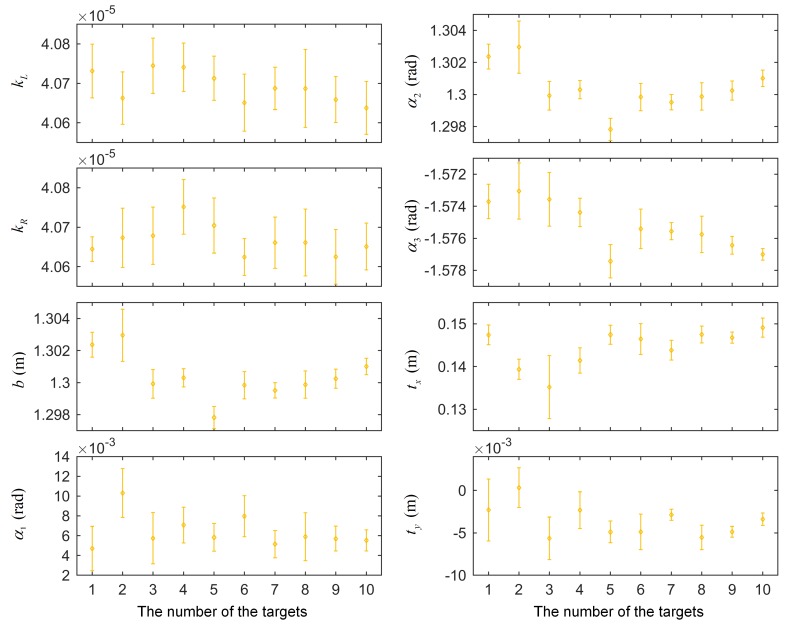
Calibration results with different numbers of the targets.

**Figure 14:**
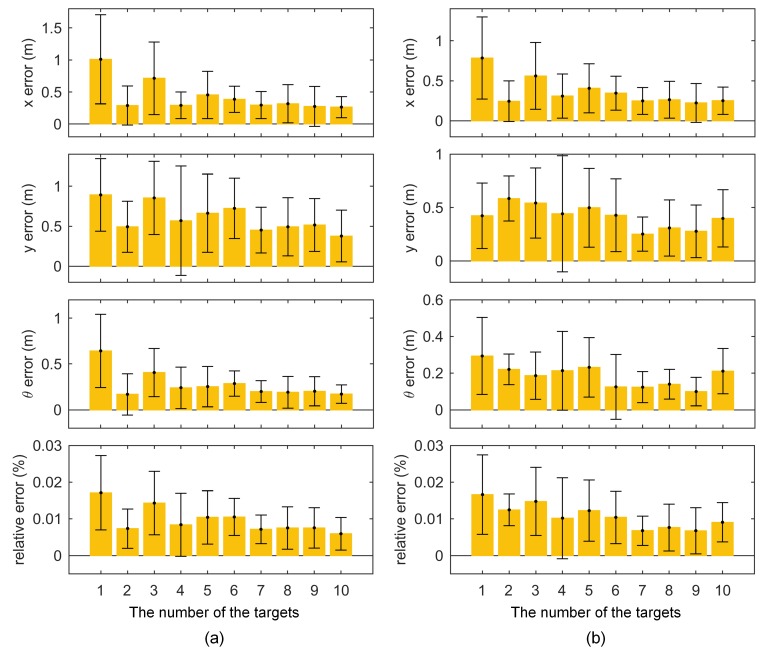
Odometry errors with different number of the targets under trajectories (**a**,**b**) from Figure 8.

To test the impact of the number of targets on the performance of the proposed method, 1–10 targets were used and arranged as presented in Figure 12. For each form, the proposed method was run ten times. Results are shown in Figure 13 with the standard deviations. As the number of targets increased, the calibration result became more stable, the jitter of the means became smaller, and the standard deviations were reduced. The indirect odometry calibration accuracy test method used in Section 4.3 was also used here with results shown in Figure 14. Overall, as the number of targets increased, the odometry error exhibited a downward trend. However, some abnormal situations appeared, presumably because (1) the trajectories of the 10 pieces of data were identical; (2) the number of targets observed by the keyframes were different; and (3) the moving speeds of the robot varied, causing different motion blurs among the 10 data and introducing different noises.

### 4.6. Design Odometry Parameters Comparison

The odometry parameters calibrated by the proposed method were also compared with the design odometry parameters. The left/right wheel factor $k_{L/R}$ can be obtained by the robot’s mechanical parameters:

(31) $$k_{L/R}=\frac{2\pi r_{L/R}}{N_{L/R}I_{L/R}},$$

where $r_{L/R}$ is left/right radius, $N_{L/R}$ is pulse number of a round of the left/right encoder that is mounted on the shaft of the left/right motor, and $I_{L/R}$ is the reduction ratio of the left/right motor. Using the designed parameters shown in Section 4.1, we get the design odometry parameters: parameters: $r_{L/R}=3.7410\times 10^{-5}$, b=0.32 m. The compared odometry parameters calibrated by the proposed method were obtained from the data in front of the target placement form C in Section 4.2. The mean results of data C1–C3 were used: $k_{L}=4.0652\times 10^{-5}$, $k_{R}=4.0668\times 10^{-5}$, and b=0.3166.

**Figure 15:**
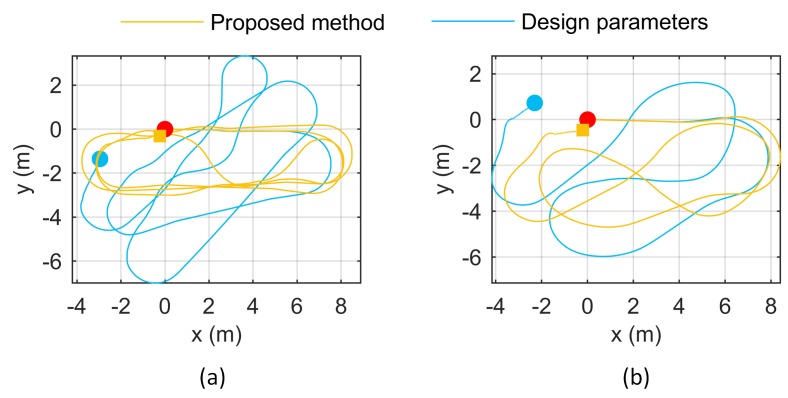
Odometry trajectories using design odometry parameters and calibrated odometry parameters under data (**a**,**b**) of Section 4.3.

The two sets of odometry parameters were compared using the method in Section 4.3. These two sets of odometry parameters were loaded into the robot respectively, and the two sets of data in Section 4.3 were run. The obtained trajectories are shown in Figure 15. It can be seen that, under the design odometry parameters, the odometry trajectories quickly diverge. In contrast, the odometry parameters calibrated by the proposed method enable high accuracy odometry trajectories.

## 5. Discussion

Our approach is similar to the work of [15]. The authors used multiple aruco markers to estimate camera poses and designed a two-step pipeline to simultaneously calibrate the odometry and the robot extrinsic parameters. First, the initial parameters are estimated through a non-iterative process by exploiting plane motion constraints; then, the parameters are refined by a joint optimization. This method has been proven to be robust to image noises and requires only a few aruco markers to be arranged in the environment, and is simple to operate. However, the method has its own limitations. First, one of the problems of aruco markers is that the accuracy of their corner positions is not too high, even after applying sub-pixel refinement, which results in a low estimation of the camera poses; moreover, the effect of the number of the aruco markers on the calibration result is not tested. Second, the plane constraint is only introduced into the initial value estimation process, but is not into the join optimization solution step; in addition, only the simulation experiment is carried out, but no real experiment is performed to verify the role of the plane constraint. In contrast, the proposed method uses multiple composite targets, which combine the advantages of the aruco marker and the checkerboard pattern. The corners of checkerboard patterns can be refined more accurately since each corner is surrounded by two black squares, which results in a more precision calibration result than that of the aruco marker. It has been tested by our experiments. In addition, an automatic pipeline to combine these composite targets to select keyframes and estimate keyframe poses is proposed. In addition, we design an experiment to test the impact of the number of the composite targets. Moreover, we introduce two types of planar constraints and use them in all calibration processes. In the end, the effects of planar constraints were tested experimentally.

We have to point out that the proposed method uses multiple composite targets that are a bit complicated to fabricate and arrange. In contrast, the natural feature-based method [12,13,14] does not require targets or other equipment, and is thus easier to use. However, it also has its own limitations. For example, it requires sufficient texture in the environment, which means that the calibration results may vary in different environments. Moreover, the monocular camera pose estimated by the visual simultaneous localization and mapping method or the structure from motion method is up to a scale; the scale is usually obtained by pre-calibrated odometry parameters [13]. In this paper, the purpose of using multiple composite targets is to improve the stability and accuracy of the calibration. Of course, using fewer targets can reduce the complexity of the calibration, but the calibration accuracy and stability will reduce. In practical applications, a balanced selection can be made between the number of targets and the calibration accuracy and stability.

## 6. Conclusions

In this paper, we propose an automatic pipeline to simultaneously estimate the odometry and robot extrinsic parameters of a differential-drive mobile robot equipped with a monocular camera. This approach does not limit the path of the robot and does not require an initial assumption of calibration parameters, producing relatively more accurate and stable results than other methods. To address the low-accuracy problem of traditional artificial landmark tools, we propose a composite target consisting of an aruco marker and a checkerboard pattern and introduce a method to automatically combine multiple composite targets, select keyframes, and estimate keyframe poses. Initial values of the calibration parameters are computed by an analytical method and then optimized via two optimization steps. Several experiments were conducted to test the stability and accuracy of the proposed approach as well as the effectiveness and roles of key strategies. Results confirm the comparable performance of this method.

## Figures and Tables

**Table 1:** Rectangle errors of the four different methods. The bold number indicates that the error is minimum.

Error Term	Proposed	NoPlanar	Analytical	Aruco
length (m)	0.0005	0.0011	0.0119	**0.0003**
width (m)	0.0026	0.0029	**0.0016**	0.0075
angle (rad)	**0.0077**	0.0096	0.0612	0.0127
